# Public health round-up

**DOI:** 10.2471/BLT.15.010815

**Published:** 2015-08-01

**WHO delivers medicines and clean water in Yemen**

WHO and its health partners are distributing medicines and other life-saving health assistance in conflict-torn Yemen, as well as clean water. This photograph shows a WHO water consignment for 985 displaced families in the districts of Saqyn (Sa'ada Governorate) and Khamer (Amran Governorate).

**Figure:**
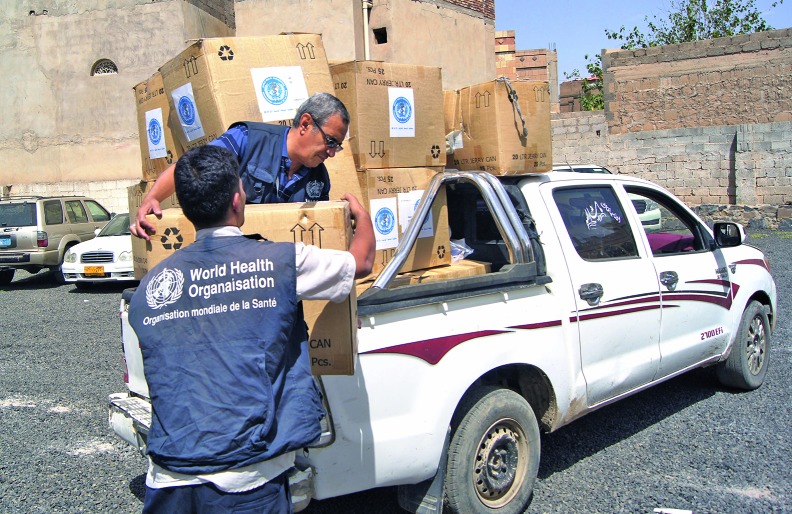
WHO/Sadeq Al-Wesabi

## IHR Review Committee to discuss Ebola report 

Dr Margaret Chan, WHO Director-General, will convene the first meeting of the newly formed International Health Regulations (IHR) Review Committee on the Ebola outbreak 24 to 25 August in Geneva. 

The Review Committee, comprising experts from WHO Member States, will discuss recommendations by the Ebola Interim Assessment Panel in its final report, released on 7 July, on the response by WHO and the international humanitarian system to the current Ebola outbreak in West Africa.

The IHR (2005) are rules on how WHO’s Member States should handle disease outbreaks and other health emergencies.

The Committee will assess how effective the regulations have been during the current Ebola outbreak and the extent to which 2011 recommendations, made by the IHR Review Committee on the 2009 influenza pandemic, have been implemented.

In its report, the Ebola Interim Assessment Panel called for significant changes at all levels of the Organization and in Member States, so that WHO could “better perform its core function of protecting global health”.

The panel recommended several measures, including a 5% increase in WHO’s regular budget and the establishment of a new WHO Centre for Emergency Preparedness and Response, integrating the outbreak control and humanitarian areas of work that are currently separate.

The panel called on WHO’s Member States and partners to contribute immediately to a contingency fund for outbreak response, with a target of US$ 100 million.

A WHO statement said: “WHO is already moving forward on some of the panel’s recommendations including the development of the global health emergency workforce and the contingency fund to ensure the necessary resources are available to mount an initial response.” 

http://www.who.int/csr/disease/ebola/panel-to-assess-response/

## Tobacco taxes

Raising excise taxes on cigarettes and other tobacco products is one of the most effective and cost-effective ways to reduce smoking, but few countries levy these at optimal levels, according to a new WHO report.

Only 33 countries currently impose taxes of more than 75% of the retail price of cigarettes, while many countries have extremely low cigarette tax rates and some have none at all, according to the WHO *Report on the global tobacco epidemic 2015*.

The report was launched in the Philippines in recognition of the country’s efforts to reduce smoking by tripling national excise taxes in 2012.

It is the latest in a WHO report series that tracks tobacco control progress in countries by gauging their implementation of key policies based on WHO’s so-called MPOWER measures, of which raising tobacco taxes is one.

In 2008, WHO identified these as the evidence-based measures that are the most effective in reducing tobacco use, and started to provide technical support to help countries use the measures to fulfil some of their obligations under the WHO Framework Convention on Tobacco Control.

“Evidence from countries such as France shows that higher tobacco product prices linked to increased taxes lead to significant declines in smoking and tobacco-related harm, such as lung cancer deaths,” said Dr Douglas Bettcher, director of WHO’s Department for the Prevention of Noncommunicable diseases.

In addition to raising tobacco taxes, the other MPOWER measures are: monitoring tobacco use and prevention policies; protecting people from tobacco smoke; offering help to quit tobacco use; warning people about the dangers of tobacco; enforcing bans on tobacco advertising, promotion and sponsorship.

http://www.who.int/tobacco/global_report/2015/report/

## Financing future health

WHO Director-General Dr Margaret Chan called on countries to scale up their health investments as they move towards universal health coverage at the Third United Nations Financing for Development Conference in Addis Ababa, Ethiopia, last month.

The conference looked at how the international community can boost its cooperation with low- and lower-middle income countries to combine domestic and external funding to build and maintain robust health systems.

Next month governments are due to adopt the new Sustainable Development Goals (SDGs) at the United Nations General Assembly in New York.

One of the proposed 17 goals is on health (Goal 3) and this goal comprises 13 targets, including one on universal health coverage.

A recent WHO–World Bank Group report studied global access to essential health services – including family planning, antenatal care, skilled birth attendance, child immunization, antiretroviral therapy, tuberculosis treatment, and access to clean water and sanitation – in 2013, and found that an estimated 400 million people lacked access to at least one of these types of services.

The *Tracking universal health coverage: first global monitoring report *found that, across 37 countries, 6% of the population was tipped or pushed further into extreme poverty (living on US$ 1.25/day) because they had to pay for health services out of their own pockets, the report said.

In 2013, 32% of global health expenditure came from out-of-pocket payments, down from 36% in 2000, the report noted.

“While this is the right direction, the 2013 figure is nevertheless considered an indication that in many countries out-of-pocket payments are still too high (below 20% of total health expenditure is usually a good indication of reduced risk of catastrophic health spending),” said the report.

WHO and the World Bank Group recommend that countries pursuing universal health coverage should aim to achieve a minimum of 80% population coverage of essential health services, and that everyone should be protected from catastrophic health spending.

http://apps.who.int/iris/bitstream/10665/174536/1/9789241564977_eng.pdf?ua=1

**Cover photo**

Market vendors in District 5 of Ho Chi Minh City, Viet Nam, selling a selection of fresh local produce including fish, sea food and vegetables. This photograph is one of many from all over the world illustrating the theme of this year’s Expo Milano 2015 exhibition, “Feeding the planet, energy for life”.

**Figure:**
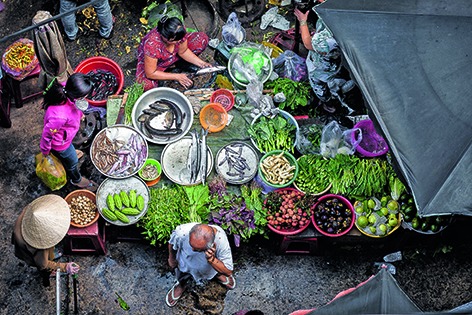
© Sylvain Marcelle

## Mental health atlas

Many low- and middle-income countries do not have enough mental-health care providers and thus fall short of universal coverage of mental health services as envisaged in WHO’s *Comprehensive mental health action plan 2013–2020*, according to a new report.

Huge inequalities in access to mental health services exist depending on where people live, according to the *WHO Mental health atlas 2014* released last month.

In high-income countries, there is one mental health worker for every 2000 people, but in low- and middle-income countries levels fall to less than one per 100 000 people.

“More primary care staff need to be trained in mental health care, so that they can recognize and treat people with severe and common mental disorders,” said Dr Shekhar Saxena, director of the Department of Mental Health and Substance Abuse at WHO.

The number of nurses working in mental health has increased by about one third since 2011, but there are still shortages in all mental health disciplines and particularly in low and middle-income countries, according to the report.

WHO’s *Mental health atlas* provides the baseline data to measure progress countries have made on the action plan targets.

The 2014 edition is the fourth and most recent edition, with data on mental health services and resources across the world, including financial allocations, human resources and specialized facilities for mental health from 171 countries.

http://www.who.int/mental_health

## WHO prize

The Thalassaemia International Federation was awarded WHO’s Dr Lee Jong-Wook Memorial Prize for its contribution to public health at this year’s World Health Assembly.

Thalassaemia is a group of genetic disorders affecting the oxygen-carrying protein, haemoglobin. It was originally thought to be a disease of the Mediterranean region, but later it was recognized that these conditions – which include sickle cell anaemia – are prevalent in areas where malaria is or was endemic.

The nongovernmental organization, established in 1987 in Cyprus has helped to set up 117 national associations in 57 countries and is working for equal access and quality of health care for all those with these conditions.

**Looking ahead**

25–27 September – United Nations Summit to adopt the post-2015 development agenda. New York, United States of America.

